# Long noncoding RNA small nucleolar RNA host gene 12/microRNA-138-5p/nuclear factor I/B regulates neuronal apoptosis, inflammatory response, and oxidative stress in Parkinson’s disease

**DOI:** 10.1080/21655979.2021.2005928

**Published:** 2021-12-16

**Authors:** Lei Yan, Lei Li, Jingan Lei

**Affiliations:** a Pain Department, The Central Hospital of Wuhan, Tongji Medical College, Hua Zhong University of Science and Technology, Wuhan, China; b Rehabilitation Medical Department, The Central Hospital of Wuhan, Tongji Medical College, Hua Zhong University of Science and Technology, Wuhan, China; c Neurology Department, Shenzhen Bao'an District Songgang People's Hospital, Shenzhen, China

**Keywords:** LncRNA SNHG12, miRNA-138-5p, NFIB, Parkinson’s disease, apoptosis, inflammation

## Abstract

Parkinson’s disease (PD) is a progressive neurodegenerative disorder that causes tremors, gait rigidity, and hypokinesia. We determined the effects of long noncoding RNA small nucleolar RNA host gene 12 (lncRNA SNHG12) on the development of PD. StarBase analysis and dual-luciferase reporter assay verified the interaction between lncRNA SNHG12 and microRNA-138-5p (miR-138-5p). The effects of suppressed lncRNA SNHG12 and increased miR-138-5p levels on mRNA were determined using quantitative real-time-PCR (qRT-PCR) in 1-methyl-4-phenylpyridinium (MPP^+^) treated SH-SY5Y cells. Increased lactate dehydrogenase (LDH) activity, apoptosis, cleaved-Caspase3/Caspase3 ratio, inflammatory response, reactive oxygen species (ROS) level, decreased cell viability, and superoxide dismutase (SOD) activity were observed in MPP^+^-stimulated SH-SY5Y cells. Transfection of the lncRNA SNHG12-plasmid reduced neuronal apoptosis, inflammation, and oxidative stress in MPP^+^-stimulated SH-SY5Y cells that were rescued by adding the miR-138-5p mimic. These results showed that lncRNA SNHG12 could affect neuronal apoptosis, inflammation, and oxidative stress in a PD cell model by regulating miR-138-5p expression. TargetScan and dual-luciferase reporter analysis suggested that miR-138-5p targeted nuclear factor I/B (NFIB). Furthermore, the expression level of NFIB was downregulated after MPP^+^ stimulation in SH-SY5Y cells. After transfecting with the miR-138-5p inhibitor, NFIB-siRNA, and co-transfecting and detecting NFIB mRNA and protein, we found that miR-138-5p negatively regulated NFIB expression. In conclusion, lncRNA SNHG12 could alleviate neuronal apoptosis, inflammation, and oxidative stress in a PD cell model by regulating the miR-138-5p/NFIB axis, providing new therapeutic targets for patients with PD.

## 1. Introduction

PD is a progressive neurodegenerative disease that causes tremors, gait rigidity, and hypokinesia [1,2]. It occurs mainly because of degenerating neurons in the substantia nigra pars compacta (SNpc) and the formation of fibrillar, α-synuclein-containing Lewy body intracytoplasmic inclusions [3,4]. The pathology of PD is complex and has not been completely explained. Aging, oxidative stress, inflammation, and cell apoptosis are all factors associated with the pathogenesis of PD [5–8].

lncRNAs are involved in regulating gene expression at transcriptional, post-transcriptional, and epigenetic levels [9,10], and an increasing number of studies confirm that lncRNAs participate in almost every cellular process. The expression of lncRNAs is dysregulated in human diseases, such as cancer [11,12]. Recent studies have shown that lncRNA plays an important role in the occurrence and development of PD [13,14]. And lncRNAs have been reported to serve as putative biomarkers and therapeutic targets for PD [15]. LncRNA SNHG12―located in the p35.3 region of chromosome 1―is an autophagy inducer. It is neuroprotective and might offer a possible strategy for the diagnosis and treatment of multiple cancers [16,17]. However, its specific role and mechanism in patients with PD require further investigation.

Studies have shown that upregulation of miR-138-5p inhibits Mn-treated autophagy, involved in neurodegenerative diseases [18]. Downregulation of miR-217/138-5p suppresses inflammation and oxidative stress and induces neuronal apoptosis by regulating sirtuin 1 in a PD cell model [19]. In addition, miR-138-5p regulates colorectal cancer migration and chemoresistance via the NFIB-Snail1 axis [20].

Bioinformatics analysis revealed a direct binding site between lncRNA SNHG12 and miR-138-5p. We also found that NFIB directly interacts with miR-138-5p. We hypothesized that lncRNA SNHG12 might regulate PD via the miR-138-5p/NFIB axis. Therefore, this study was designed to reveal the role of lncRNA SNHG12 in MPP^+^ induced PD *in vitro* model and explore its underlying mechanisms.

## 2. Materials and methods

### 2.1 Cell culture and transfection

Human embryonic kidney 293 T (HEK293T) cells and SH-SY5Y cells were purchased from the Stem Cell Bank and Chinese Academy of Science (Shanghai, China). The cells were cultured in DMEM (Gibco, USA) supplemented with 10% FBS (Gibco, CA, USA) and 1% penicillin/streptomycin at 37°C in a humidified incubator with 5% (v/v) CO_2_.

To simulate PD, SH-SY5Y cells were first exposed to various concentrations (0, 0.25, 0.5, 1, and 2 mM) of MPP^+^ (Sigma, St. Louis, MO, USA) for 24 h, or stimulated with MPP^+^ (1 mM) for various periods (0, 6, 12, 24, 48 h), and used as PD models.

Mimic control (5′‐GCGGUCGUGCAGUGCGUGAUAUA‐3′; RiboBio Inc.), miR-138-5p mimic (5′‐AGCUGGUGUUGUGAAUCAGGCCG‐3′; RiboBio Inc.), control-siRNA (cat. no. sc-36,869; Santa Cruz Biotechnology), and NFIB-siRNA (cat. no. sc-43,561; Santa Cruz Biotechnology) were transfected into SH-SY5Y cells for 48 h. Meanwhile, SH-SY5Y cells were transfected with control-plasmid, lncRNA SNHG12-plasmid, inhibitor control (5′-CAGUACUUUUGUGUAGUACAA-3′; RiboBio Inc.), miR-138-5p inhibitor (5′-CGGCCUGATTCACAACACCAGCT-3′; RiboBio Inc.), lncRNA SNHG12-plasmid + control mimic, lncRNA SNHG12 plasmid + miR-138-5p mimic, miR-138-5p inhibitor + control-siRNA, or miR-138-5p inhibitor + NFIB-siRNA for 48 h with or without the following 24 h of stimulation with 1 mM MPP^+^. Lipofectamine 2000 reagent was used for the transfection.

### 2.2 Dual-luciferase reporter assay

The targets of lncRNA SNHG12 and miR-138-5p were predicted using bioinformatics software (StarBase) and Dual-Luciferase Reporter Assay System (Promega). To confirm the association between lncRNA SNHG12 and miR-138-5p, the fragments of lncRNA SNHG12, including the miR-138-5p binding sites, and the fragments with mutant miR-138-5p-binding sites were cloned into pmirGLO (Promega, Madison, WI, USA). Then, HEK293T cells were cultured in 24-well plates and co-transfected with miR-138-5p or control mimic and luciferase reporter plasmids 24 h later. Subsequently, the latent interacting sites between NFIB and miR-138-5p were predicted using TargetScan. To confirm the association of NFIB with miR-138-5p, the wild-type and the mutant of the 3ʹ-untranslated region (3ʹ-UTR) of NFIB were cloned into pmirGLO. 293 Ts were cultured in 24-well plates and co-transfected with miR-138-5p or miR-control and luciferase reporter plasmids 24 h later. After transfection for 48 h, luciferase activity was checked using the Dual-Luciferase Reporter Assay System [21].

### 2.3 RNA isolation and qRT-PCR

Total RNA was extracted using TRIzol reagent (Invitrogen). qRT-PCR was conducted using an ABI 7300 Fast real-time PCR system and SYBR Green Master mix to determine the relative expression levels of lncRNA SNHG12, miR-138-5p, and NFIB mRNA. miRNA and mRNA expression levels were normalized to U6 and GAPDH, respectively. The following primers for *β*-actin, U6, lncRNA SNHG12, miR-138-5p, and NFIB (Sangon Biotech, Shanghai, China) were used:

*β*-actin-forward, 5ʹ-CGTGGAACTGGCAGAAGAGG-3ʹ;

*β*-actin-reverse, 5ʹ-GGAATGAGAAGAGGCTGAGACA-3ʹ;

U6-forward, 5ʹ-GCTTCGGCAGCACATATACTAAAAT-3ʹ;

U6-reverse, 5′-CGCTTCACGAATTTGCGTGTCAT-3ʹ;

lncRNA SNHG12-forward, 5′-GAAAAAGCACACCAGCTATTGG-3ʹ;

lncRNA SNHG12-reverse, 5′-CGGGATCTCTGTAGACTAAGTCAGT-3ʹ;

miR-138-5p-forward, 5′-GCGAGCTGGTGTTGTGAATC-3ʹ;

miR-138-5p-reverse, 5′-AGTGCAGGGTCCGAGGTATT-3ʹ;

NFIB-forward, 5′-TGAGGCAGCTTCACCTACAG-3ʹ;

NFIB-reverse, 5′-AGGATGGGTCTCTTGGGCTTA-3ʹ. Target gene expression was quantified using the 2^−ΔΔCt^ method [22].

### 2.4 3-(4,5-dimethylthiazol-2-yl)-2,5-dipheyltetrazolium bromide (MTT) assay *[23]*

After inoculating and precipitating SH-SY5Y cells in 96-well plates, MPP⁺ (1 mM) was added to the wells. After 24 h, SH-SY5Y cells were cultured with 10 μl of MTT solution (Sigma) for 4 h. The OD was measured at 570 nm using a microplate spectrophotometer.

### 2.5 Flow cytometry (FCM) assay *[24]*

Apoptosis was detected using a double-staining apoptosis detection kit (Annexin V-FITC Apoptosis Detection Kit; Beyotime) [24]. SH-SY5Y cells were cultured in 6-well plates and incubated overnight at 37°C and 5% CO_2_. Cells were collected after transfection and centrifuged at 1000 × g at 4°C for 5 min. After that, the cell suspension was incubated with 100 μl FITC-binding buffer, 5 μl ready-to-use Annexin V-FITC, and 5 μl PI at room temperature in the dark for 30 min. Cell apoptosis was assessed within 1 h by flow cytometry using a FACSCalibur flow cytometer (Becton Dickinson). Data were analyzed using the FlowJo software (version 7.6.1; FlowJo LLC).

### 2.6 Western blotting *[25]*

Total concentrations of extracted proteins were determined using the BCA Protein Assay Kit (Thermo Fisher Scientific). The same amounts of protein were mixed with SDS loading buffer, separated by SDS-PAGE, and transferred onto PVDF membranes, followed by blocking with 5% skimmed milk for 1 h, and then cultured with primary antibodies: GAPDH (37kDa; 1:1,000; cat. no. 5174; Cell Signaling Technology, Inc.), caspase3 (35 kDa; 1:1,000; cat. no. 14,220; Cell Signaling Technology, Inc.), cleaved-caspase3 (19 kDa; 1:1,000; cat. no. 9654; Cell Signaling Technology, Inc.), Bax (20 kDa; 1:1,000; cat. no. 5023; Cell Signaling Technology, Inc.), Bcl-2 (26 kDa; 1:1,000; cat. no. 15,071; Cell Signaling Technology, Inc.), and NFIB (47 kDa; 1:1,000; cat. no. 186,738; Abcam) at 4°C overnight. The following morning, after washing with PBST, the membranes were incubated with the secondary antibodies (Cell Signaling Technology, Inc.) for 1 h at RT. Finally, protein bands were visualized using ECL detection system reagents (Cell Signaling Technology) as per the manufacturer’s instructions. Proteins were normalized to GAPDH.

### 2.7 Lactate dehydrogenase (LDH) release assay *[13]*

LDH is an indicator of cell death. SH-SY5Y cells were treated with (MPP⁺, 1 mM). After 24 h incubation, the activity of LDH was determined using an LDH Cytotoxicity Assay Kit (cat. no. C0016; Beyotime Biotechnology, Shanghai, China). OD_490_ was detected using a microplate reader.

### 2.8 Enzyme linked immunosorbent assay (ELISA) *[13]*

The concentration of TNF-α (cat. no. PT518) and IL-1β (cat. no. PI305) secreted into the medium by MPP^+^-stimulated SH-SY5Y cells was determined using ELISA according to the manufacturer’s protocol (Beyotime Biotechnology, Shanghai, China). The OD_450_ was measured using an ELISA plate reader.

### 2.9 Reactive oxygen species (ROS) assay *[19]*

The ROS assay kit is based on the oxidation and subsequent change in fluorescence intensity of 2, 7-dichlorodi-hydrofluorescein diacetate (DCFH-DA) in the presence of ROS. It is the most commonly used method for the quantitative determination of intracellular ROS levels. SH-SY5Y cells stimulated with MPP^+^ were incubated with 100 μM DCFH-DA (Yeasen, Shanghai, China) at 37°C in the dark for 30 min. Fluorescence intensity was determined using a microplate reader according to the manufacturer’s instructions.

### 2.10 SOD activity *[19]*

SH-SY5Y cells were washed, harvested, centrifuged, then the SOD activity in cell supernatant was detected using the SOD activity assay kit (cat. no. S0109; Beyotime Biotechnology, Shanghai, China) following the manufacturer’s instructions.

### 2.11 Statistical analysis

All experiments were conducted in triplicate. Results were analyzed statistically using GraphPad Prism software (version 7.0; GraphPad) and expressed as the mean ± standard deviation (SD). Comparisons among groups were performed using Student’s *t*-test or one-way analysis of variance (ANOVA) followed by Tukey’s test. Statistical significance was set at *P* < 0.05.

## 3. Results

### 3.1 lncRNA SNHG12 interacts with miR-138-5p

**Figure 1.**
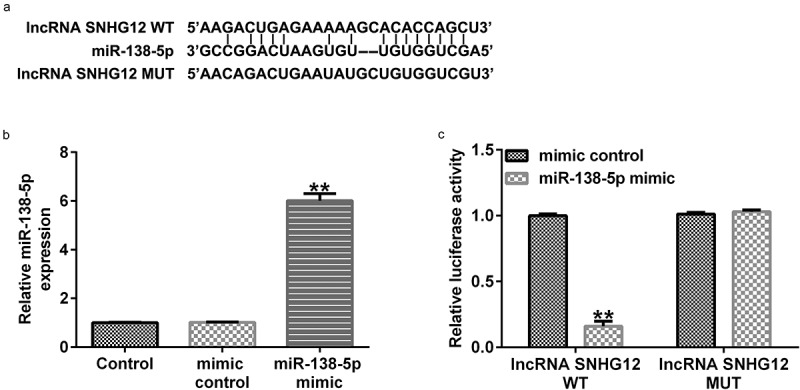
lncRNA SNHG12 interacts with miR-138-5p. (a) StarBase software was used to predict the possible target of lncRNA SNHG12, namely miR-138-5p, and their binding sites. (b) miR-138-5p levels in control mimic- or miR-138-5p mimic-transfected 293 T cells. (c) The binding sites between lncRNA SNHG12 and miR-138-5p were verified using dual-luciferase reporter analysis

We first determined the relationship between lncRNA SNHG12 and miR-138-5p. Starbase analysis showed that lncRNA SNHG12 directly interacts with miR-138-5p at its binding sites (Figure 1a). We confirmed this interaction using a dual-luciferase reporter analysis. miR-138-5p mimic enhanced miR-138-5p levels in 293 T cells (Figure 1b) and decreased the luciferase activity of lncRNA SNHG12-WT compared with the control mimic group (Figure 1c). There was no obvious change in the luciferase activity in cells transfected with the MUT-lncRNA SNHG12 3ʹ-UTR reporter plasmid (Figure 1c). These findings illustrate that lncRNA SNHG12 targets miR-138-5p.

### 3.2 MPP^+^ stimulation decreased lncRNA SNHG12 expression and increased miR-138-5p levels in SH-SY5Y cells

**Figure 2.**
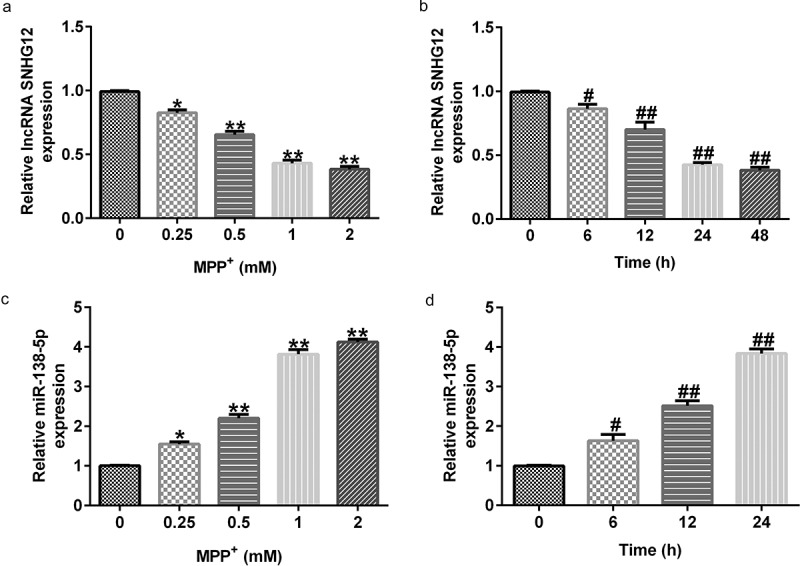
Levels of lncRNA SNHG12 and miR-138-5p in PD cell model. qRT-PCR analysis of lncRNA SNHG12 (a, b) and miR-138-5p (c, d) in SH-SY5Y cells stimulated with MPP^+^ at different doses or for various times

Having illustrated the relationship between lncRNA SNHG12 and miR-138-5p, we further determined their expression using qRT-PCR in SH-SY5Y cells stimulated with various concentrations of MPP^+^ for 24 h or exposed to MPP⁺ (1 mM) for specified times. MPP^+^ treatment significantly decreased lncRNA SNHG12 expression in SH-SY5Y cells in a dose- and time-dependent manner (Figure 2a and b) but increases the miR-138-5p levels (Figure 2c and d). These results suggest that lncRNA SNHG12 and miR-138-5p might be negatively correlated.

### 3.3 SNHG12 negatively regulated the expression of miR-138-5p in SH-SY5Y cells

**Figure 3.**
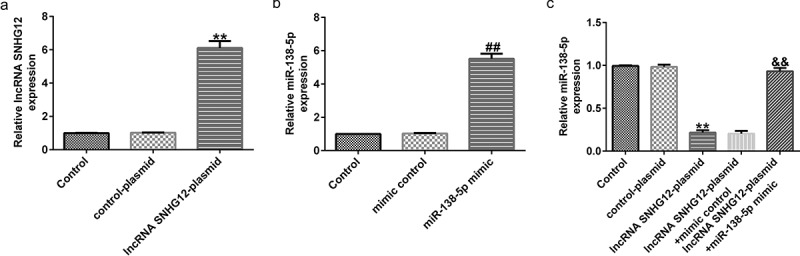
Effects of lncRNA SNHG12 on miR-138-5p expression in SH-SY5Y cells. SH-SY5Y cells were transfected with the control mimic, miR-138-5p mimic, control-plasmid, or lncRNA SNHG12-plasmid. Determination of lncRNA SNHG12 expression (a) and miR-138-5p levels (b, c) in different groups using qRT-PCR analysis

To explore whether lncRNA SNHG12 is involved in the progression of PD via the miR-138-5p axis, six groups of transfected SH-SY5Y cells (control plasmid, lncRNA SNHG12-plasmid, control mimic, miR-138-5p mimic, lncRNA SNHG12-plasmid + control mimic, and lncRNA SNHG12-plasmid + miR-138-5p mimic) were prepared. qRT-PCR analysis revealed enhanced expression of lncRNA SNHG12 in lncRNA SNHG12-plasmid transfected SH-SY5Y cells (Figure 3a and b). Similarly, the expression of miR-138-5p was increased after miR-138-5p mimic transfection compared to the control plasmid group. The results in Figure 3c show dramatically reduced levels of miR-138-5p after lncRNA SNHG12-plasmid transfection. The effect was eliminated after co-transfection with the lncRNA SNHG12-plasmid and miR-138-5p mimic.

### 3.4 The lncRNA SNHG12 reduced neuronal apoptosis, inflammation, and oxidative stress in an in vitro PD cell model

**Figure 4.**
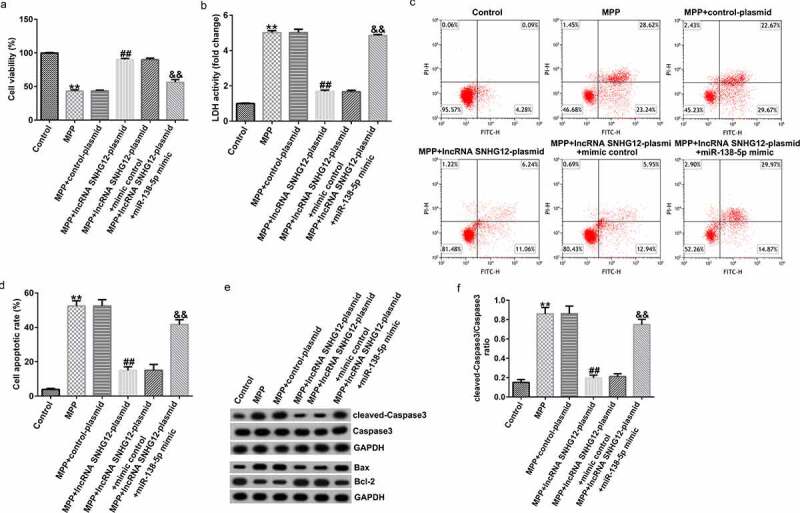
Effects of lncRNA SNHG12 on cell viability and apoptosis in *in vitro* PD cell model. The control plasmid, lncRNA SNHG12-plasmid, lncRNA SNHG12-plasmid + control mimic, and lncRNA SNHG12-plasmid + miR-138-5p mimic were transfected into SH-SY5Y cells for 48 h following 24 h of stimulation with 1 mM MPP^+^. Cell viability (a), LDH activity (b), and apoptotic rate (c and d) were measured using MTT, LDH release, and FCM analysis, respectively. (e) Expression of cleaved-Caspase3, Caspase3, Bax, and Bcl-2 detected by western blot assay. (f) Determination of cleaved-Caspase3/Caspase3 ratio

To further investigate the functional roles of lncRNA SNHG12 and miR-138-5p in the PD model, we transfected the cells with a control plasmid, lncRNA SNHG12-plasmid, lncRNA SNHG12-plasmid + control mimic, and lncRNA SNHG12-plasmid + miR-138-5p mimic for 48 h after stimulating them with 1 mM MPP^+^ for 24 h. Compared with the control group, the cell viability markedly decreased after stimulation with MPP^+^. Treating the cells with lncRNA SNHG12-plasmid significantly increased the cell viability compared with the control plasmid. The effect was reversed by co-transfection with the miR-138-5p mimic (Figure 4a). In addition, the LDH release assay (Figure 4b) showed increased LDH release in MPP^+^-stimulated SH-SY5Y cells compared with the control. In contrast, lncRNA SNHG12 reduced the LDH activity, which was rescued by co-transfection with the miR-138-5p mimic. FCM was used to analyze apoptosis in SH-SY5Y cells. MPP^+^ treatment induced apoptosis (Figure 4c and d), while transfection with the lncRNA SNHG12-plasmid reduced it. However, the miR-138-5p mimic reversed this situation. Western blot suggested that MPP^+^ upregulated cleaved-Caspase3, enhanced Bax, reduced Bcl-2 expression, and increased the ratio of cleaved-Caspase3/Caspase3. We observed an opposite trend in the lncRNA SNHG12-plasmid group (downregulated cleaved-Caspase3, decreased Bax, increased Bcl-2 expression, and decreased cleaved-Caspase3/Caspase3 ratio) where the cells were rescued by co-transfection with miR-138-5p mimic (Figure 4e and f).

**Figure 5.**
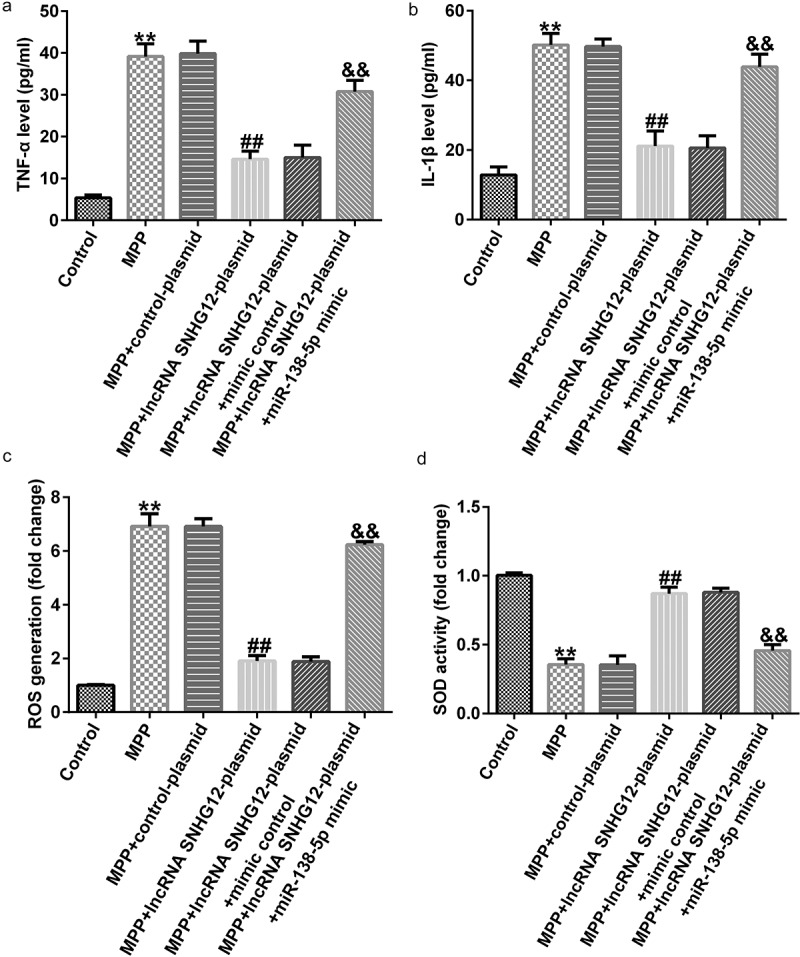
Effects of lncRNA SNHG12 on inflammation and oxidative stress in PD cell model. SH-SY5Y cells were transfected with the control plasmid, lncRNA SNHG12-plasmid, lncRNA SNHG12-plasmid + control mimic, and lncRNA SNHG12-plasmid + miR-138-5p mimic for 48 h following 24 h of stimulation with 1 mM MPP^+^. Levels of TNF-α (a) and IL-1β (b) were measured using ELISA. Detection of ROS release (c) and SOD activity (d) in SH-SY5Y cells

ELISA results showed enhanced TNF-α and IL-1β secretion after MPP^+^ stimulation that was further improved after lncRNA SNHG12-plasmid treatment. Co-transfection with the miR-138-5p mimic reversed this effect (Figure 5a and b). Moreover, the effect of lncRNA SNHG12 and miR-138-5p on oxidative stress was determined by ROS release and SOD activity assays. This demonstrated that MPP^+^ administration significantly increased ROS release and decreased SOD activity relative to the control. lncRNA SNHG12 weakened these effects, and co-transfection with miR-138-5p mimic rescued them (Figure 5c and d). In conclusion, our findings illustrated that lncRNA SNHG12 could reduce neuronal apoptosis, inflammation, and oxidative stress in MPP^+^-induced SH-SY5Y cells by regulating miR-138-5p.

### 3.5 NFIB directly targets miR-138-5p

**Figure 6.**
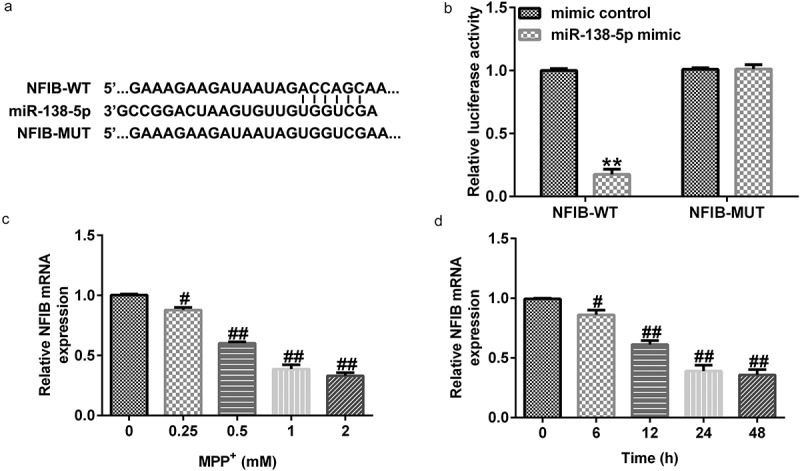
miR-138-5p directly targets NFIB and regulates its expression in *in vitro* PD cell model. (a) The TargetScan software predicted the possible target of miR-138-5p, namely NFIB, and their binding sites. (b) The binding sites between miR-138-5p and NFIB were verified using dual-luciferase reporter assay. (c, d) qRT-PCR analysis of NFIB in SH-SY5Y cells treated with MPP^+^ at different doses or for different times

To determine the possible mechanism of miR-138-5p in PD, we identified its possible gene targets using TargetScan. miR-138-5p has a potential binding site on the NFIB 3ʹ-UTR (Figure 6a). Dual-luciferase activity assay demonstrated that luciferase activity of WT-NFIB-3ʹUTR reporter plasmid was inhibited by adding miR-138-5p mimic. There were no significant changes in luciferase activity in the MUT-NFIB group (Figure 6b). These results verified the interaction between NFIB and miR-138-5p.

### 3.6 MPP^+^ stimulation decreased NFIB expression in SH-SY5Y cells

To determine the impact of NFIB in PD, we treated SH-SY5Y cells with different concentrations of MPP^+^ for 24 h or stimulated them with 1 mM MPP^+^ for different times. The expression levels of NFIB were examined using qRT-PCR. Our results suggest that MPP^+^ decreased NFIB expression in a dose- and time-dependent manner in SH-SY5Y cells (Figure 6c and d).

### 3.7 miR-138-5p inhibition upregulated NFIB expression in SH-SY5Y cells

**Figure 7.**
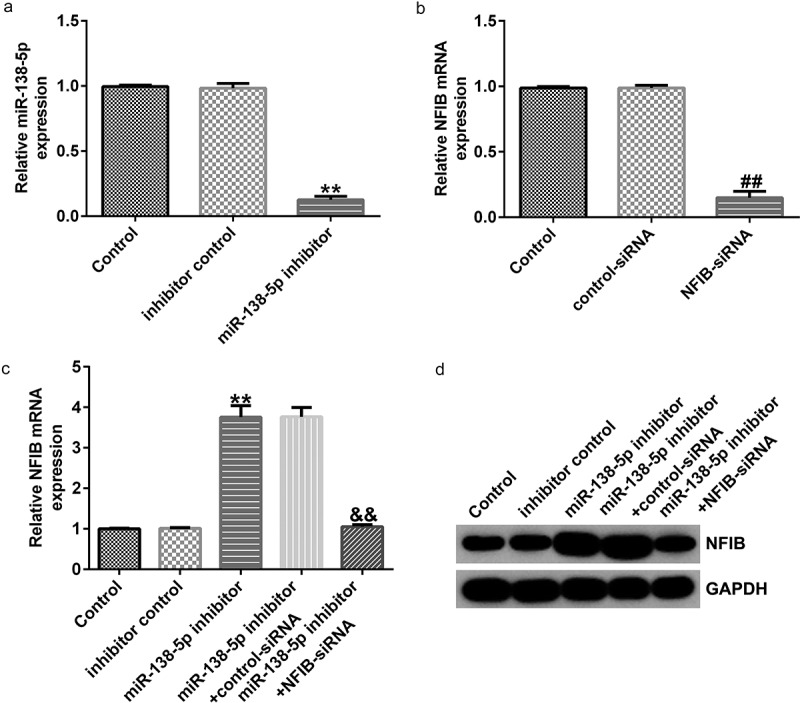
Effects of miR-138-5p inhibitor on NFIB levels in SH-SY5Y cells. SH-SY5Y cells were transfected with the control inhibitor, miR-138-5p inhibitor, control-siRNA, or NFIB-siRNA. Expression of miR-138-5p (a) and NFIB (b) was examined using qRT-PCR in SH-SY5Y cells. qRT-PCR and western blot analysis (c and d) of NFIB expression in SH-SY5Y cells

To confirm the regulatory effect of miR-138-5p on NFIB in PD, control inhibitor, miR-138-5p inhibitor, control-siRNA, and NFIB-siRNA were transfected into SH-SY5Y cells. We found that miR-138-5p levels were remarkably reduced in miR-138-5p inhibitor-transfected SH-SY5Y cells compared to the inhibitor control (Figure 7a), while NFIB was downregulated in SH-SY5Y cells treated with NFIB-siRNA (Figure 7b). The expression of NFIB mRNA and its protein levels were remarkably improved after transfection with miR-138-5p inhibitor compared with the inhibitor control (Figure 7c and d). However, this effect was eliminated in SH-SY5Y cells by co-transfection with NFIB-siRNA.

### 3.8 Downregulation of miR-138-5p inhibited neuronal apoptosis, inflammation, and oxidative stress in a PD cell model

**Figure 8.**
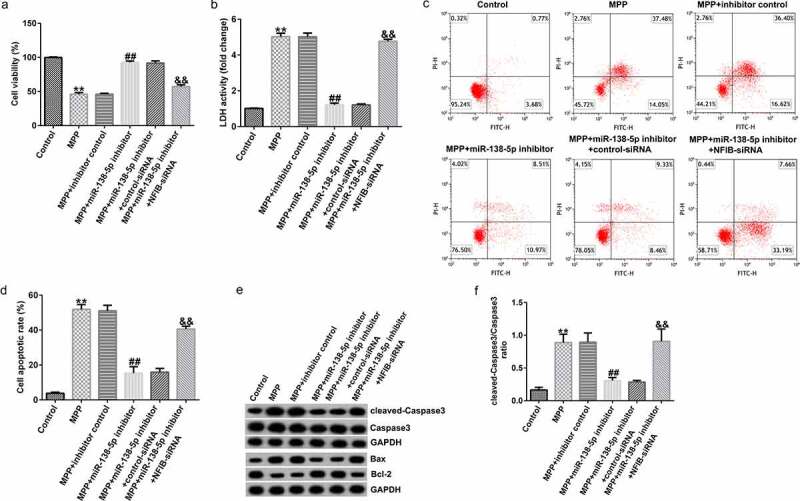
Effects of miR-138-5p inhibitor on SH-SY5Y cell viability and apoptosis. SH-SY5Y cells were transfected with the control inhibitor, miR-138-5p inhibitor, miR-138-5p inhibitor + control-siRNA, and miR-138-5p inhibitor + NFIB-siRNA for 48 h following 24 h of stimulation with 1 mM MPP^+^. Cell viability (a), LDH activity (b), and apoptosis rate (c and d) were measured using MTT, LDH release, and FCM analysis, respectively. (e) Expression of cleaved-Caspase3, Caspase3, Bax, and Bcl-2 detected using western blot assay. (f) Determination of cleaved-Caspase3/Caspase3 ratio

To determine the interaction between miR-138-5p and NFIB, SH-SY5Y cells were transfected with the control inhibitor, miR-138-5p inhibitor, control-siRNA, and NFIB-siRNA for 48 h after treating them with 1 mM MPP^+^ for 24 h. Results from MTT and FCM assays indicated that MPP^+^-induced decrease in cell viability and LDH activity and increase in cell apoptosis were reversed by the iR-138-5p inhibitor. The effects of MPP⁺ were restored by NFIB-siRNA treatment (Figure 8a-d). In addition, we observed that miR-138-5p inhibitor reduced cleaved-Caspase3 expression, decreased Bax expression, enhanced Bcl-2 expression, and reduced cleaved-Caspase3/Caspase3 ratio in MPP^+^ -stimulated SH-SY5Y cells. This effect was rescued after NFIB-siRNA treatment (Figure 8e and f).

**Figure 9.**
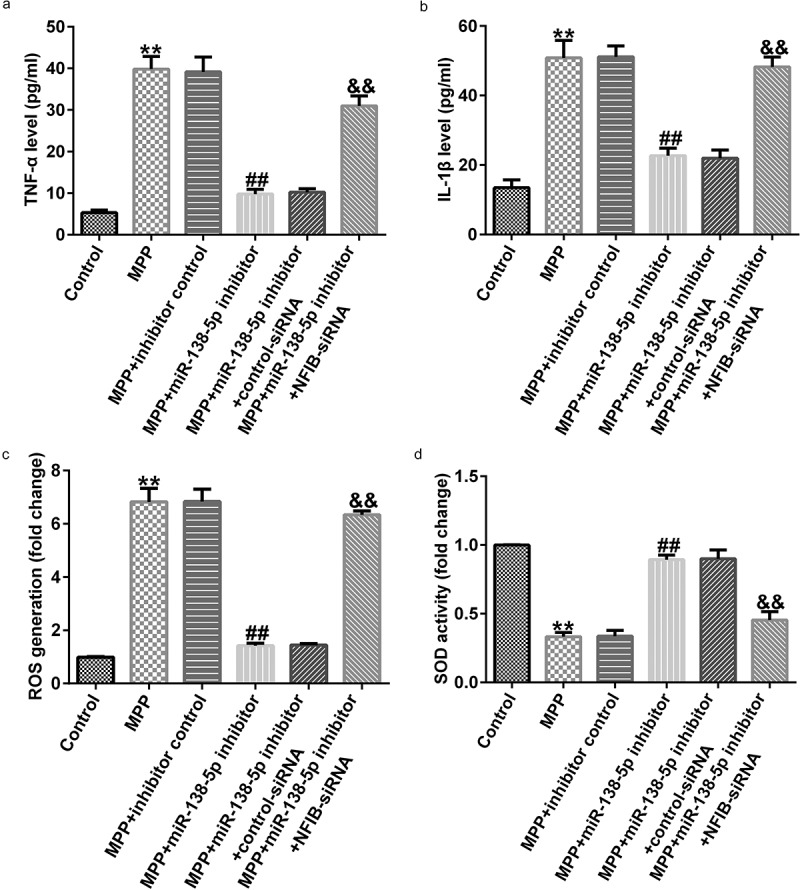
Effects of miR-138-5p inhibitor on inflammation and oxidative stress in PD cell model. SH-SY5Y cells were transfected with the control inhibitor, miR-138-5p inhibitor, miR-138-5p inhibitor + control-siRNA, and miR-138-5p inhibitor + NFIB-siRNA for 48 h following 24 h of stimulation with 1 mM MPP^+^. The secretion of TNF-α (a) and IL-1β (b) was measured using ELISA. Determination of ROS release (c) and SOD activity (d) in SH-SY5Y cells

We also illustrated the functions of miR-138-5p inhibitor in inflammation in a PD cell model using ELISA. TNF-α and IL-1β levels were significantly decreased in MPP^+^-treated SH-SY5Y cells treated with miR-138-5p inhibitor. The cells were rescued by co-transfection with NFIB-siRNA (Figure 9a and b). Inhibition of miR-138-5p decreased the ROS release and increased SOD activity in MPP^+^-treated SH-SY5Y cells, and these effects were reversed by treating the cells with NFIB-siRNA (Figure 9c and d).

## 4. Discussion

PD neuropathology includes a selective lack of dopaminergic neurons in the SNpc, affecting other CNS structures and peripheral tissues. Various factors―environmental, epigenetic, and genomic―are related to PD pathology and change the conformation and deposition of key proteins due to dysfunction of the ubiquitin proteasome and mitochondria and oxidative stress [26–30]. The lncRNA SNHG12 could relieve injury in SH-SY5Y cells after OGD/R by activating autophagy [17]. It is involved in the progression of neuronal apoptosis as it interacts with disease-associated miRNAs [31]. In our study, we verified the interaction of lncRNA SNHG12 with miR-138-5p using the StarBase database and dual-luciferase reporter assay.

miRNAs are essential regulatory factors involved in the progression of various diseases, including PD. miR-138-5p causes neuronal apoptosis, inflammation, and oxidative stress by targeting sirtuin 1 in MPP^+^-stimulated SH-SY5Y cells [19]. We tested the binding between miR-138-5p and NFIB using TargetScan and a dual-luciferase reporter assay. A previous study reported that individuals haploinsufficient for NFIB showed muscle tone regression, motor and language deficits, attention deficit disorder, autism spectrum disorder, and behavioral abnormalities [32].

Our results demonstrated that MPP^+^ treatment reduced lncRNA SNHG12 expression in a dose- and time-dependent manner, and enhanced miR-138-5p expression. However, levels of lncRNA SNHG12 and miR-138-5p in PD cell model detected by Northern blot will make the data more convincing. Meanwhile, lncRNA SNHG12 negatively regulated miR-138-5p expression in SH-SY5Y cells. Experiments confirmed that the induction of lncRNA SNHG12 markedly increased cell viability and decreased apoptosis and LDH activity. LDH is a stable cytoplasmic enzyme. Damage to the plasma membrane―the main characteristic of apoptosis, necrosis, and other forms of cell damage―leads to the release of enzymes into the medium, including LDH [33]. It should be noted that although flow cytometry assay with Annexin V-FITC apoptosis detection kit is a recognized method for apoptosis detection, the Tunel method is the most reliable method for apoptosis detection. This study only used flow cytometry assay detects cell apoptosis, which is another limitation of this study. Previous studies revealed high levels of pro-inflammatory regulators in the midbrain of patients with PD [34]. According to our ELISA results, upregulation of lncRNA SNHG12 decreased the high expression levels of TNF-α and IL-1β caused by MPP^+^ treatment. Furthermore, we measured the negative effect of lncRNA SNHG12 on oxidative stress in the cell model using ROS release and SOD activity assays. However, all the effects caused by lncRNA SNHG12 were reversed by miR-138-5p. These experiments illustrate that lncRNA SNHG12 suppresses neuronal apoptosis, inflammation, and oxidative stress by downregulating miR-138-5p. Using the same methods, we further confirmed the decreased NFIB expression in MPP^+^-stimulated SH-SY5Y cells and the negative regulation of NFIB by miR-138-5p that resulted in increased neuronal apoptosis, inflammation, and oxidative stress in the PD model.

It should be noted that this study is only a preliminary *in vitro* study of the role of lncRNA SNHG12 in PD. In order to make the role of lncRNA SNHG12 in PD more convincing, more in-depth research is needed. For example, it is necessary to explore the role of lncRNA SNHG12 in other PD cell models. The functional role of NFIB on lncRNA SNHG12 reducing neuronal apoptosis, inflammation, and oxidative stress in PD cell model needs to be further clarified. Besides, the lncRNA SNHG12 level in PD patients and *in vivo* models should be verified. Moreover, the role of lncRNA SNHG12 in PD animal models also need to be explored. We will perform these issues in the future.

## 5. Conclusion

LncRNA SNHG12 contributes to neuronal apoptosis, inflammation, and oxidative stress in a PD cell model by regulating the miR-138-5p/NFIB axis, providing possible therapeutic targets for PD.

## Data Availability

The datasets used and/or analyzed during the current study are available from the corresponding author upon reasonable request.

## Research highlights

- lncRNA SNHG12 interacts with miR-138-5p.
- The lncRNA SNHG12 reduced neuronal apoptosis, inflammation, and oxidative stress in an in vitro PD cell model.
- Downregulation of miR-138-5p inhibited neuronal apoptosis, inflammation, and oxidative stress in a PD cell model.
